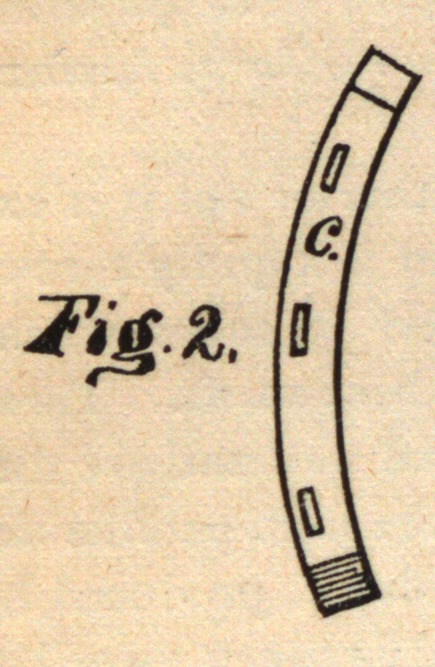# The Expansion and Contraction of the Horse’s Foot, and How to Shoe It

**Published:** 1882-07

**Authors:** John N. Navin


### ART. XV.—THE EXPANSION AND CONTRACTION OF THE HORSE'S FOOT, AND HOW TO SHOE IT.

BY JOHN N. NAVIN, V.S.

(*Continued.*)

How far I have succeeded in proving that the foot expands and contracts in violent action, that its expansion obviates concussion of its internal sensitive parts, and that the elasticity of the laminæ, and arched form of the sole, and not the frog, contribute to its required expansion and contraction the reader must be his own judge. It does seem to me, indeed, that no man possessing common anatomical or mechanical skill can fail to perceive that a soft elastic organ, such as a healthy frog, is inadequate to the mechanical force requisite to keep a stout wall apart, and at the same time overcome the resistance necessary to lowering the arched form of the sole, which it must do in forcing the wall outward. How much more seemly it is, that the weight of the horse thrown upon the crown of the arched sole, would force its base outwards, thereby expanding the wall, than that a wedge of any kind (which the frog is claimed to be) would, whilst joined firmly to the sole, force the wall apart. I care not what kind of substance the frog may be composed of, be it hard or soft, whilst firmly united to the sole, it has a tendency to push that organ upward, and increase its concave or arched form, therefore contributing more to contracting than expanding the foot when in violent action it strikes the ground.

That the wall expands and contracts no man seems to deny. The entire controversy seems to hinge upon its mode of expansion. Such being the facts, the reader will naturally inquire what kind of shoe, if not the one at present in use, is the one to adopt.

The answer naturally suggests itself. A shoe which gives full protection from wear and tear and permits full and free expansion and contraction of the wall, as the unshod foot possesses.

This inquiry naturally leads to another, viz.: How is the wall constructed, and by what organ or organs is it secreted? On no subject pertaining to the anatomy and physiology of the horse has there been a greater diversity of opinion than that of the production of the wall, from the earliest period to the present day. Some highly educated veterinarians contend that the tubular portion of the foot is secreted by the coronary border (*which is correct*), but that the connecting matrix and insensible laminæ are secreted by the sensible laminæ; others admit that the insensible laminæ are secreted by the sensible, but that the connecting matrix is secreted by the coronary.

For over twenty-seven years I had been floating between these two theories, having heard lectures and read books advocating both doctrines, until an independent idea struck me, more than twenty years ago, and I concluded to satisfy my mind upon the subject. I therefore went to work to dissect specimens, and no sooner had I established an independent opinion of my own than I cowed under that written by some eminent veterinarian, and feared to assert my own simple opinion against that of distinguished writers; therefore, no sooner would I finish writing up the subject than, when contrasting my theories with those of scientists, my manuscript was consigned to the flames, and I found myself humiliated by my own want of courage. One thought, however, I never could smother, and that was that both theories could not be correct. I therefore concluded that I would contend for my own, which differed from both, and that if the high reputation of either crushed me to the wall, I would be no more annihilated than the third party. So, with fear on one side and ambition on the other, I searched and researched until I satisfied myself that the coronary border secretes not only the tubular portion of the wall, but also the matrix and insensible laminæ, and intend to prove it.

In placing before the public a few of the conflicting opinions of veterinarians, upon the formation and secretion of the wall of the foot, and how and by what organ or organs it is secreted, I beg to call attention to the theory of a distinguished and flowery writer, viz., Joseph Bruce Coleman, M.R.C.V.S., Chicago. In his work on "Pathological Horse-Shoeing," which embraces the anatomy and physiology of the foot, page 39, in speaking of the functions of the coronary ligament, he says: "The locale of the ligament is in the coronary concavity, just inside the superior margin of the wall; it is the principal medium of connection between the skin and hoof; it appears to be transitional in its character, and partaking of the characters of both. Its surface towards the wall is papillated, and each papilla penetrates a horn tube or fibre, of which the fibrous portion of the wall is composed. It is affirmed by one writer (Mr. Fleming) that the entire wall is secreted by this ligament; this, of course, would include the horny laminæ." I dissent from this opinion for the following reasons: The wall is composed of two kinds of material, the fibrous and non-fibrous matrix, which bind the fibres firmly into the solid mass of horn, which constitutes the wall of the foot. I am not aware of any secreting structure which secretes more than one specific secretion in a healthy acting condition. But if there should be, it would not be sufficient reason for attributing the functions of secreting two such widely different substances as compose the walls to the coronary ligament. Each fibre of the wall is fed by its connecting papilla; besides, we are not left in doubt as to where the matrix comes from. I think it is just as demonstrable as the source of the horny fibres. The vascular laminæ have not merely a mechanical connection with the non-vascular horny laminæ, but a secreting function, and it is from this source that the homogeneous or non-fibrous portion of the wall is derived. If an experimental proof of this is demanded, examples are numerous enough, in cases of false quarter and those cases where the hoof has been torn by violence, and the coronary ligament partially or wholly destroyed. In false quarter, for instance, when the coronary ligament is injured or destroyed, we have but a thin non-fibrous horny secretion composing the quarters, frequently too weak to sustain the weight of the animal, which could come from no other source than the vascular laminæ. Reason—*Versus ipse dixit.* In cases where the entire hoof has been torn off, whilst the vascular laminæ remain otherwise uninjured, a non-fibrous horny material will be deposited all over the sensitive laminæ in a few weeks. Does this come from the coronary ligament? It cannot, as ten or twelve months will scarcely suffice for the growth of horn, from the top to the bottom of the foot. I think these experimental facts too well known to need any further exemplification. Mr. Fleming, in his recent work on horse-shoeing, asserts that the latter is an error, as the microscope, physiology and pathological experience abundantly testify. This is all the refutation he has condescended to give this alleged error. Supposing it to be an error? It can hardly be expected that those accustomed to do their own thinking will weigh the opinions of others for themselves and to rely on their own pathological experience, when they have any, will readily abandon their own opinion, without a single reason being advanced, or a particle of evidence being adduced, to show that they are erroneous, beyond the mere *ipse dixit* of any writer, however he may be an authority in other respects. I repeat, the principal function of the coronary ligament is to secrete the fibrous portion of the wall. It is extremely vascular and highly elastic. The former quality is necessary to supply the demand of rapid wear and tear, to which the wall is specially subject in the unshod foot, and to maintain the integrity of the structure it secretes, the latter to adapt it to the expansive and contractile movements of the foot when in action, or when weight is superimposed upon or removed from the limb. Although in appearance a thickening of the terminal portion of the skin, it has a peculiar and distinct organization, to adapt it to its very important purposes. The inner layer is a strong white elastic substance, which forms its principal thickness: upon this reposes a numerous accumulation of blood vessels, and external to these a thin coat, from which proceed the secreting villi of the walls. The attachments of these villi in the living structure must be of a powerful nature, as there is no visible connection between the hoof and the skin, excepting the areolar or connective tissue which bind it, but in no especial manner, to all the adjacent structures. The growth of the wall is in every respect similar to the growth of the human nail.

Dr. Dadd, V.S., in his work, pages 28–29, says: "The sensitive laminæ make no addition to the substance or thickness of the wall; they simply produce the horny laminæ along its interior, and as a proof of which, the wall measures as much in the thickness at the place where it quits the coronet as at any other point lower down. Other demonstrations of this fact come every day before such practitioners as have to treat canker, quittor, sand-crack, and other diseases of the feet." Again, at page 30, under the title of "The Coronary Substance," he says: "To revert, for sake of elucidation here, to former description. After the hoof has been detached by a process of maceration or putrefaction, in a perfectly entire and uninjured condition, it presents around its summit a circular groove, bounded in front by a soft whitish substance, having a thin edge, and having a nature between horn and cuticle, and behind by an attenuated margin, more horny in its character, whose thin edge is serrated or denticulated. Into this circular groove or canal is received the terminal margin of the cutis, the cuticulo-horny layer of the hoof in front of it having every appearance of being a continuation of the cuticle. The coronary substance occupies the coronary concavity, formed upon the inside of the superior or coronary border of the wall of the hoof. It is the part constituting the basis of the circular prominence commonly distinguished in the living animal as the coronet. It is broader around the toe of the wall, diminishing in breadth towards the quarters and heels, and being somewhat broader around the outer than the inner side. It is thicker in substance around the middle and most prominent parts, growing gradually thinner both above and below." In page 31, he says: "The coronary substance may be ranked amongst the most vascular parts of the body. No gland even possesses, for its magnitude, a greater abundance of blood-vessels, or of blood-vessels of a larger size, nor does there any part exist in which greater care is taken to arrange its vessels so as to insure an uninterrupted supply of blood. *These vessels it is that produce the wall*, and, there is every reason to believe, without any assistance from the vessels of the laminæ." Again, on page 33, writing on the organization of the sensitive laminæ, he says: "All the blood they have occasion for is that which is sufficient only for the secretion of the horny laminæ."

Another distinguished author, Prof. Williams, in his work, page 352, speaking of false quarter: "The horny wall or crust of the foot, being secreted by the coronary substance, it naturally follows that, when part of it is destroyed, the part of the wall below the destroyed portion is no longer supplied with horn from above, and this deficiency causes a chasm or fissure in the wall. It differs from sand-crack very materially, is much wider at its base, and contains a modified condition of horn, viz., the horny laminæ secreted by the sensitive ones, denuded of their outer covering. The coronary band also shows a loss of substance, corresponding to that in the crust."

Again he says, in page 354, quoting that great anatomist, Barlow: "The horn tubes are cemented together by an intertubular substance, composed of cells, and produced from the surface between the papillæ. The wall, consisting of tubes and agglutinating intertubular substance, is secreted by the coronary substance, and is naturally tough. Having seen, then, that the horn is secreted by the papillæ and interpapillary substance, the reader can understand that the brittle condition of the crust, leading to sand-crack, depends upon a perverted condition of the secretory structures."

I find myself widely differing from the theory of each of the above gentlemen, and I shall therefore offer a few arguments and proofs in support of my own, which is, that the horny tubes, connecting matrix, and the horny insensitive laminæ are each secreted, produced by the coronary ligament, and I think I can prove it to every man of intelligent, perceptive mind. The merest novice will not deny that when the coronary band is divided by violence, it throws out (secretes) a divided wall; then, no matter how small the crack may be, the sensitive laminæ never yet was known to furnish matrix sufficient to unite the edges of the crack; but no sooner is a hot chissel-edged instrument forced through the wall at its junction with the inferior edge of the coronary border, severing the old wall from the border, than the effect of the heated iron swells the tissues, its edges meet, heal, and produce an undivided hoof. This cannot be denied or disproven. I have done it a thousand times. I, like others, have been simple enough to clasp cracked hoofs, but not lately, nor shall I ever do so again. What becomes of those gentlemen's theories, in case where those useful appendages, the lateral cartilages, become inflamed, ossified and enlarged, reaching above and behind the coronet? Does their covering membrane still continue the secretion of the matrix? If so, where does it go to? Certainly not to the hoof, although under those altered conditions the wall appears perfectly formed and fully secreted. Will the advocates of those foolish theories inform me whether the superior part of the laminæ secretes the matrix, or if their whole surfaces, from coronary to sole, do so? If the latter, why does a sand-crack remain divided until it grows down completely? If their theory is correct, it should be filled by the matrix at any point that it touches the laminæ. I shall then refer the reader to both these theories, which vary from my own (which is that the wall, matrix and insensible laminæ are secreted from the coronary substance), and proceed to give a few illustrations to prove my correctness.

Will these gentlemen inform us that the sensitive laminæ upon the lateral cartilage secrets the insensible laminæ and connecting matrix at their superior junction? If they do, away goes their theory. When the ossified cartilage extends behind and above the coronary ligament, it surely cannot secrete in front of the ligament; and if they say that they secrete along their entire length, away goes their theory in cases of laminites, in which the dovetailed junction existing between the sensitive and insensitive laminæ is severed, and the coffin-bone descends upon the sole. Still in both cases the wall continues to be secreted by the coronary border.

I was first prompted to inquire into this matter by observing that white-legged horses generally have white hoofs, but should they happen to have a black spot in the region of the coronet, a black streak in the hoof below it invariably follows, and *vice versa.*

This is easily accounted for, when we consider the fact, that the middle layer of the skin in which the hair bulbs repose controls the color of the hair, and in white-colored horses the cuticle also. If, then, the coronary is a prolongation of the skin, why should not the complete wall, both its matrix and tubular part, be a prolongation of the coronary. Some time since I treated a black mare suffering from an injury to her coronet and pastern. She had been treated by an empiric; and when I saw her the part was much inflamed and swollen, the coronet divided longitudinally by some sharp body. On reduction of the inflammation, I remarked a shell-like body curling upwards from the coronet; and I found that organ divided in its centre longitudinally about one inch in length, the outer division turned upward, and secreting a well-developed hoof, which I cut down, but the secretion continued, and a subsequent growth revealed a well-developed hoof, presenting both a matrix and tubular secretion. This illustration completely disproves all other theories which cannot be proven on practical observation and experience. When a quittor passes between the wall and os pedis, from sole to coronary, destroying the laminæ, or at least completely severing their connection, will any one tell me the tubular part of the wall alone is secreted under those circumstances? Again, if the sensible laminæ secrete the matrix, how does it find its way through the wall itself, in order to supply its outer part, it being at least three-fourths of an inch in thickness? Again, how is the matrix of the superior and upper portion of the coronary concavity secreted? Surely not by the laminæ, from which it is completely disconnected, but from the coronary above, to which it is immediately connected. I myself have passed a seton through from sole to coronet, and allowed it to remain for some time, and of course severing the laminæ. Where did the matrix come from, then, to supply the wall? Although its substance appeared perfect and continued so for months under my own personal observation. About a year since I treated a case of fistula of the coronet. On introducing the probe, it passed right from the sole to the coronet in an indirect line from above, downwards and forwards. After trying various milder remedies, I removed a V-shaped portion of the wall, next the coronet, exposing the sinus. The depression representing the portion I removed, of course, remained, until it disappeared in the ordinary way, but no matrix ever appeared to fill it. If the matrix is secreted from the sensitive laminæ, why not perform their functions in this case as well as if the tubular portion were present, and fill at least a portion of the depression? Mr. Coleman argues that no organ in health maintains a double secretion, as a proof that the coronary does not secrete both tubes and matrix of the wall. Can he account for the production of the sole and frog? From what source do they derive their secretion? Surely he cannot claim that the coronet laminæ supply anything to those bodies. Again, he says: "If the entire hoof be torn off the living animal, it becomes covered with a horny secretion from the sensitive laminæ in a few weeks." But he seems to forget that nature is kind, under unlooked-for conditions, and that even bone, when exposed, becomes covered by a protecting membrane. For further convincing proof of my correctness, I refer those gentlemen to that great and well-known anatomist, Chauveau, in his work, "Comparative Anatomy," which I became possessed of only a few weeks since *for the first time*, and I was indeed highly gratified to find that I had such a distinguished author for a reference in my favor. Page 810, on the development of the hoof, he says: "The hoof being a dependency of the epidermis, is developed like it; that is, by the incessant formation of cells, in the layer that corresponds to the rete mucosum, at the expense of the plasma, thrown out by the numerous vessels in the veratogenous membrane. The velvety tissue forms the sole and frog, the perioploic ring the periople, and the coronary cushion the wall. In these different parts the epithelial cells multiply and become flattened in layers parallel to the surface that secretes them, and in proportion as they recede from the surface, so that the wall grows from its superior to its inferior border, and the other two parts of the hoof from its internal to their external face. The villiosities of the coronary cushion and the velvety tissue are the organs around which the epithelial laminæ are grouped, and their presence determines the tubular structure of the horn. Their peculiar function is completed by the exhalation of a peculiar fluid that maintains the flexibility of the hoof, and probably by the development on their surface of the irregular cells which cluster in the interior of the tubes. The leafy tissue in a normal condition *does not concur to any extent in the development of the wall.* The cells covering it are multiplied in describing a downward and forward movement, and though they are certainly applied to the inner surface of the wall, yet they do not constitute the horny laminæ. The latter are formed from the coronary cushion, at the commencement of the va cular laminæ, and they descend with the wall, in gliding along the surface of the layer of cells, separating them from the latter. This downward movement is facilitated by the mu'tiplication in the same sense of these cells. *This opinion as to the function of the vascular laminæ is based on comparative anatomy, on the presence of some longitudinal tubes in the horny laminæ, and on pathological observations.*

When the podophyllous tissue is inflamed, whether or not it is exposed, its latent activity is quickly manifested, and it rapidly throws out a quantity of hard consistent horn, traversed by tubes which, according to Mr. Gourdon, are directed obliquely backwards. These tubes are more irregular than those of the normal wall and are disposed in parallel series, and are formed around the villo-papillæ developed on the free border of the laminæ. In this horn, produced by the vascular laminæ only, there are never observed between those latter the horny plates of cells sharp and distinct, in the midst of other cells, as those in the wall formed by the coronary cushion. *The horn thrown out on the surface of the podophyllæ, immediately after removal of a fragment of the wall, is not a definite horn, but must be replaced by that from the coronet.* This substitution is complete, as a microscopical examination proves that the wall which descends from the cushion and is furnished with horny laminæ, passes beneath the provisional wall and glides downwards by the combined action already mentioned over the surface of the soft cells of the vascular laminæ, as soon as the latter are covered by the proper wall ; then marginal papillæ become atrophied, and they again assume the limited function pertaining to their physiological condition. The quotation is a splendid exposure of Mr. Coleman's so-called "secretion of horn from the sensitive laminæ when the hoof is torn off."

I inclose you the description of my shoe, as set forth in my patent claim.

The objects of my invention are, first, to provide a shoe for horses which can be so fastened to the foot as to allow free play to the natural elasticity of the walls of the hoof, and thus prevent disease of the foot; second, to afford a means of curing or relieving the disease known as "contracted hoof" by spreading the walls of the hoof after the shoe has been placed in position and retaining them in the desired position; third, to provide a convenient means for securing pads or other appliances in contact with the frog or under side of the foot. I attain these objects by the use of the shoe illustrated in the accompanying drawings.

*a* is a shoe, of the usual form, in the upper side of which is formed a dovetailed recess, *b*, into which is nicely fitted a separate removable piece, *c*, thus completing the contour of an ordinary shoe. The removable piece *c* is provided with nail-holes, as is also the other side of the shoe, in the usual manner.

*d d* are upward-projecting lugs, formed upon or welded to the upper surface of the shoe at the heel, and designed to fill loosely the space between the walls of the hoof when the shoe is first secured to the foot. A portion of the shoe is cut away at the toe of the inner edge, at *e*, for the purpose of making the shoe weaker at that point, so that when force is applied between the lugs *d d* to spread that portion of the shoe apart it will bend at that point, *e*, and not at any intermediate point, the object of this being to prevent any cramping or binding of the removable piece *c* in its recess, and also to secure an equable and proper spreading of the hoof in a case of diseased hoof, as hereinafter explained.

For the purpose of securing to the under side of the foot rubber pads or other appliances used in the treatment of diseased feet, the lugs *d d* are provided with holes *f*. By passing a small rod or wire through these lugs and the pad or other appliance before the shoe is secured to the foot the pad when applied is securely held in place.

My improved shoe is applied and operates in the following manner: The shoe being fitted to the foot in the usual manner, the position of the removable piece *c* is marked upon the hoof. It is then removed from its recess and nailed to the foot in its proper position. The shoe is then slipped upon the piece *c*, the dovetailed form of which retains that side of the shoe in place, and the other side is then nailed in the usual manner.

When the horse rests upon the foot or strikes a violent blow, as in running, the piece *c* slides in its dovetailed recess, and allows the natural elasticity of the walls of the hoof to operate, thus preventing disease.

If the foot is already in a diseased condition from wearing a stiff, unyielding shoe, and has become contracted, I now apply a suitable lever between the lugs *d d* and spread the shoe, thus bending it at *e* and spreading it permanently, mostly at the heel, diminishing gradually to the toe, and holding the walls of the hoof apart. This being done with care and good judgment leaves the softer portions of the foot free to regain their normal condition and relieves the disease.

## Figures and Tables

**Fig. 1. f1:**
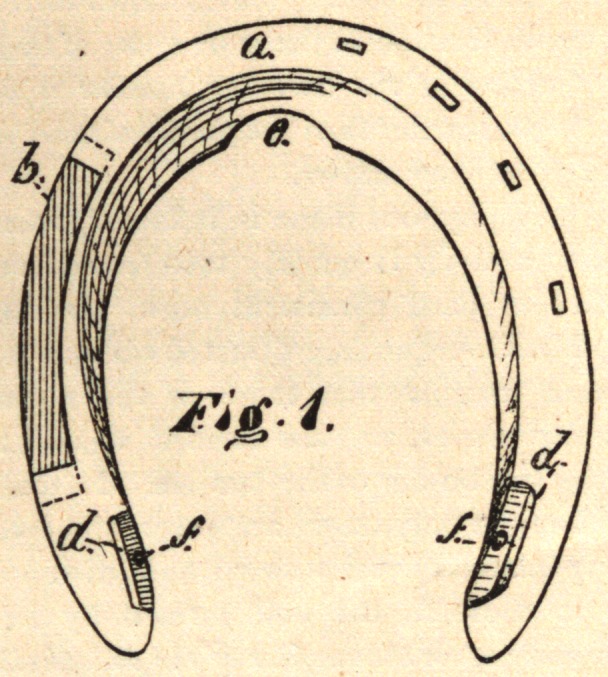


**Fig. 2. f2:**